# HHV8/EBV Coinfection Lymphoproliferative Disorder: Rare Entity with a Favorable Outcome

**DOI:** 10.1155/2017/1578429

**Published:** 2017-02-09

**Authors:** Dhouha Bacha, Beya Chelly, Houda Kilani, Lamia Charfi, Amel Douggaz, Samia Chatti, Emna Chelbi

**Affiliations:** ^1^Pathology Department, Mohamed Tahar Maamouri Hospital, Mrezga, Nabeul, Tunisia; ^2^Medicine Faculty of Tunis, Tunis El Manar University, Tunis, Tunisia

## Abstract

HHV8/EBV-associated germinotropic lymphoproliferative disorder (GLD) is a challenging diagnosis given its rarity, the particular clinical presentation, and the lack of expression of markers usually used in establishing hematopoietic lineage. We report a new case of HHV8/EBV GLD in an immunocompetent 78-year-old woman. The diagnosis was made in an incidentally discovered lymphadenopathy. Histological examination showed a nodular lymphoid proliferation centered by aggregates of atypical plasmablastic cells admixed with small lymphoid cells. Tumor cells were strongly positive with EMA, HHV8, LMP1, CD38, CD138, and kappa light chains. They were negative with common lymphoma-associated markers (CD20, CD3, CD15, CD30, CD10, and bcl2). In situ hybridization confirmed the monotypic kappa light chains and the EBV infection (EBER+). A polyclonal pattern of Ig gene rearrangement was detected by PCR analysis. In the adjacent lymph node parenchyma, some germinal centers mimicked Castleman disease. In this case, the differential diagnosis was discussed with an early stage of large B-cell lymphoma arising in HHV8-associated multicentric Castleman disease. The clinical presentation, the immunophenotype, and the molecular results helped to make the accurate diagnosis. Through the review of the nine previously reported cases in literature, we discuss the clinical and pathologic features and the differential diagnosis of HHV8/EBV GLD.

## 1. Introduction

Human herpes virus 8 (HHV8)/Epstein-Barr virus- (EBV-) associated germinotropic lymphoproliferative disorder (GLD) is a rare entity that has been described in HIV seronegative patients [1].

It has morphologic and immunophenotypic characteristics that distinguish it from the other HHV8 lymphoproliferative disorders.

The diagnosis can be difficult because of its rarity and the lack of expression of markers that are usually used by pathologists in establishing hematopoietic lineage.

To the best of our knowledge, only 9 cases have been reported in the literature hitherto [1–7].

Herein, we describe the 10th case of HHV8/EBV-associated GLD whose diagnosis was incidentally made.

## 2. Case Report

A 78-year-old woman with a medical history of cirrhosis after hepatitis C, atrial fibrillation, and right cardiac failure, was admitted in gastroenterology department for abdominal pain, body weakness, and sudden weight gain of 6 kgs within a period of two weeks.

Physical examination noted lower extremity edema and distended abdomen with fluid wave and mild tenderness to palpation. Skin exam showed a few spider telangiectasias on upper chest.

Three inguinal lymphadenopathies were incidentally discovered, measuring between 3 and 7,5 cm in their largest diameter. No hepatosplenomegaly was found. Pulmonary, cardiovascular, and neurological evaluation were normal.

Laboratory tests showed iron-deficiency anemia with hemoglobin level of 8 mg/dL and a discrete leukocytosis (wbc: 45 × 10^3^/L) with a predominance of neutrophils. Platelets were slightly decreased (100 × 10^3^/L). Liver function tests revealed hypoalbuminemia and abnormal elevation of liver enzymes, indicating the liver cirrhosis. Urea tests and sodium and potassium levels were in normal limits. HIV serology was negative.

Abdominal computed tomography (CT) scan showed a nodular liver with heterogeneous texture and moderately abundant ascites. It confirmed the absence of splenomegaly and deep lymphadenopathy.

The treatment of the liver decompensation has been initiated, including an abdominal paracentesis, close monitoring of the fluid balance, and an adequate nutrition. Medical treatment associated diuretics and antibiotics.

Excisional biopsy of the largest node was performed. It showed a farm lymph node measuring 7,5 × 4 × 1,5 cm with a whitish and focally nodular cut surface.

Specimens were fixed in 10% phosphate-buffered formaldehyde and embedded in paraffin, and sections were prepared for routine light microscopy after staining with hematoxylin and eosin (HE).

Additional sections were available for immunohistochemical analysis using the avidin-biotin complex technique and commercially available antibodies. A large panel of lymphoid, plasma, and epithelial cell (EMA, CD38, CD138, light chain kappa and lambda, HHV8 latency-associated nuclear antigen 1, AE1/AE3, CD20, CD3, cyclin D1, CD56, CD15, CD30, CD10, and bcl2) was performed. EBV infection was investigated by in situ hybridization (EBV early RNA EBER) and by immunostaining using LMP1 antibody.

For immunoglobulin gene (Ig) rearrangements, DNA from paraffin sections was amplified for the CDRIII region of the rearranged IgVH gene using a mixture of seven framework 3 (FR3) family specific primers and a consensus fluorescent primer for the JH gene.

**Figure 1:**
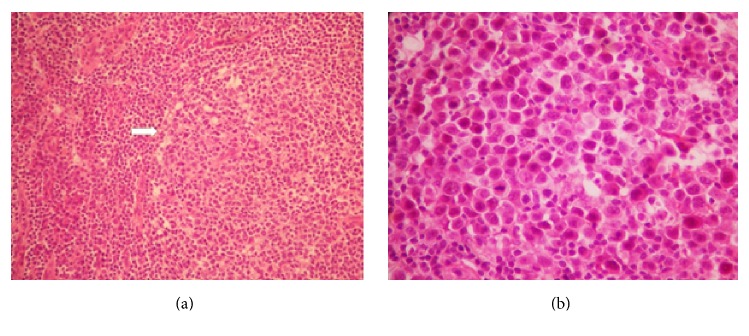
Histological examination of the lymph node. (a) Vaguely nodular lymphoid proliferation (arrow) in partially effaced architecture (HE ×100). (b) Plasmablastic cells with large eccentric nuclei, with atypical and often multilobulated contours. The cytoplasm is acidophilic and relatively abundant (HE ×400).

Histological examination showed partial effacement of the architecture by vaguely nodular lymphoid proliferation (Figure 1(a)).

Nodules were centered by aggregates of plasmablastic cells with atypical eccentric nuclei and often multilobulated contours (Figure 1(b)). Nucleoli were prominent. The cytoplasm was acidophilic and relatively abundant. These cells were also focally found in the mantle zones of residual follicles, admixed with small lymphoid cells.

**Figure 2:**
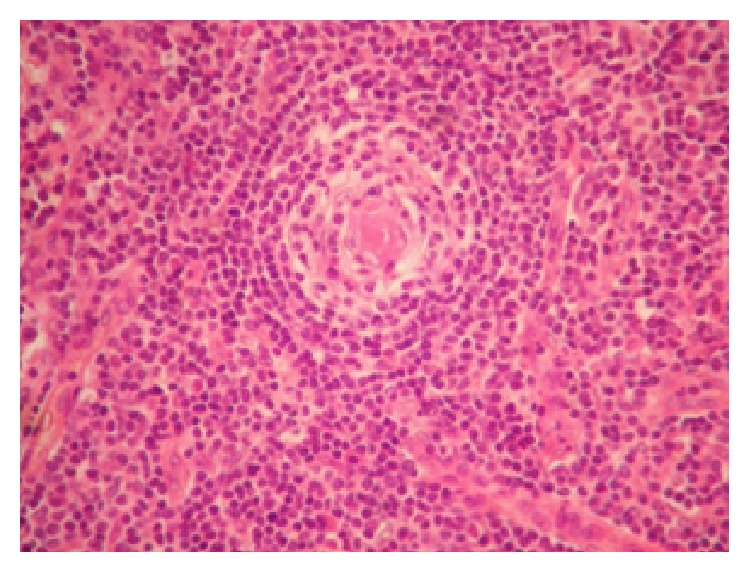
The “onion-skinning features” in some follicles of the adjacent parenchyma mimicked the Castleman disease (HE ×200).

In the adjacent lymph node parenchyma, small lymphocytes of some mantle zones were frequently arranged in concentric rings around the germinal center which was penetrated by a blood vessel “onion-skinning features,” mimicking the Castleman disease (Figure 2).

**Figure 3:**
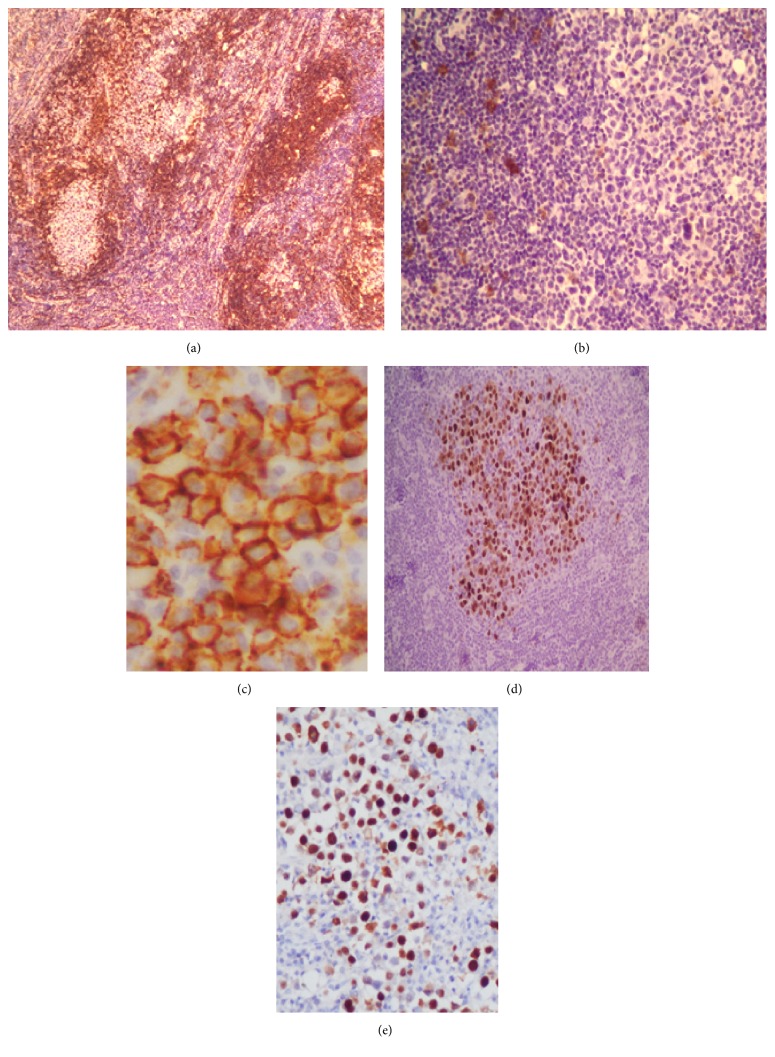
HHV8/EBV-associated germinotropic lymphoproliferative disorder: large lymphoid cells showed negative immunostaining for CD20 (magnification ×25) (a) and CD30 (magnification ×100) (b). Intense immunostaining for CD38 (magnification ×200) (c) and HHV8 (magnification ×100) (d); EBER in situ hybridization positivity of many tumor cells (magnification ×200) (e).

Immunohistochemistry showed that tumor cells were positive for EMA, LMP1, plasma cell markers (CD38, syndecan-CD138), HHV8, and kappa light chains. They were negative for the other tested antibodies (AE1/AE3, CD20, CD3, cyclin D1, CD56, CD15, CD30, CD10, and bcl2) (Figures 3(a)–3(d)). The presence of HHV8-positive cells within a meshwork of follicular dendritic cells (CD21+) confirmed the “germinal involvement” of the proliferation. In situ hybridization confirmed the monotypic kappa light chains and the EBV infection (EBER+) (Figure 3(e)). A polyclonal pattern of Ig gene rearrangement was detected by PCR analysis.

Based on the overall histologic appearance, immunophenotype, and results of molecular studies, the diagnosis of HHV8/EBV-associated germinotropic lymphoproliferative disorder was made.

Since pathological results had required a long period, the patient was discharged after the improvement of the clinical symptomatology and planned for further evaluation two weeks later. She was lost to follow-up without having any treatment.

## 3. Discussion

Human herpesvirus 8- (HHV8-) positive and Epstein-Barr virus-positive (EBV) germinotropic lymphoproliferative disorders are a rare entity, occurring typically in immunocompetent patients, not infected by human immunodeficiency virus (HIV) [1].

It is an indolent disease, which usually presents as an isolated nodal affection without general symptoms and with a favorable outcome after treatment [2].

Du et al. were the first to describe this entity by reporting 3 cases of a lymphoproliferative disease with a favorable response to chemo- and radiotherapy. They proposed the name of “KSHV and EBV-associated germinotropic lymphoproliferative disorder (GLD).” To the best of our acknowledge, only 9 cases have been documented in the literature until this date (Table 1) [1–7].

Lymphoproliferative disorders associated with HHV8 are a heterogeneous group that includes four well-defined entities: primary effusion lymphoma (PEL), multicentric Castleman disease (MCD), MCD-associated large B-cell lymphoma, and germinotropic lymphoproliferative disorder [8].

The causal relationship between HHV8 and lymphoproliferative disorder is not yet clearly elucidated [5]. According to some authors, HHV8+ lymphoproliferation may originate from B-cells at different stages of differentiation [4, 5, 7, 9, 10]. Moreover, it was suggested that the maturation stage of HHV8+ B-cells and the type of viral infection (latent or lytic) might define the pathological features of the lymphoproliferation [10].

In the literature, the review of the 9 reported cases of HHV8/EBV-associated GLD revealed a pronounced male predominance with a sex ratio of 3,5. The mean age at diagnosis was 58,6 years with a range from 41 to 75 years. All patients were HIV seronegative. In our case, the diagnosis was made in an immunocompetent woman of a more advanced age (78 years).

It is interesting to note the presence of a liver dysfunction in the personal history of our patient and in two other reported cases, with probable alcoholic cirrhosis in one case and a fatty liver infiltration in the other [3, 7]. Moreover, Du et al. reported a positive serology for hepatitis C virus in one case but we have no information about the liver function [2]. It is not clear whether the liver dysfunction at the time of diagnosis of the HHV8/EBV germinotropic lymphoproliferative disorder is an incidental finding or involved in the pathogenesis of this entity.

An isolated lymphadenopathy was the initial presentation in 6 cases [2, 4–7]. For the other cases, enlarged lymph nodes were associated with systemic symptoms in one case [1], to a leg swelling and paraesthesia in one case [2], and to ruptured aneurysm not related to the lymphoproliferative disorder in the third case [3].

The most common site of involvement is the cervical area [1–7]. Deep enlarged lymph nodes in different sites were discovered in 4 patients during staging (perirenal, paratracheal, para-aortic, and mediastinal lymphadenopathy) [2, 3].

All patients underwent lymphadenectomy. The mean size of the excisional lymph nodes was 3,65 cm with a range from 2,5 cm to 6 cm in the greatest diameter. The cut surface showed a yellow-tan, soft, fish-fleshy-like, and lobulated lesion [1–7].

Histological examination showed that the architecture could be preserved, partially or totally effaced. Tumor proliferation was composed of large cells with plasmablast's or immunoblast's morphology, disposed in small clusters, large sheets, or nodules [1–7].

They usually infiltrated the germinal centers, which were partially or completely involved. Tumor cells were also present in the mantle zone and in the interfollicular area, even without the germinal involvement. Indeed, Taris et al. reported an early form of the disease, in which the tumor cells were only noted in the mantle zone [6] and in the case of Ferry et al., atypical cells were only observed in the interfollicular zones [3]. In the majority of the cases, the diagnosis was made at an advanced stage, where germinal centers were largely replaced by the tumors cells.

The adjacent lymphoid parenchyma was rarely described. As in our case, it exhibited features of Castleman disease in one case [3] and atrophic residual lymphoid follicles in another case [1].

Given the rarity of HHV8/EBV germinotropic lymphoproliferative disorders, several antibodies were tested in all cases to confirm the final diagnosis.

The immunophenotype conventionally described is the positivity of the tumor cells for HHV8, kappa or lambda light chains, MUM1, and Ki67 in most cells. In all patients, EBV infection was found, by immunohistochemistry (LMP1) and/or EBER with in situ hybridization. Common lymphoma-associated markers are usually negative in tumor cells (CD20, CD79a, CD27, bcl6, CD10, CD 19, CD22, CD15, CD30, cyclin D1, ALK, and PAX5) as well as epithelial markers (AE1/AE3) [1–7].

Immunostaining for CD38, CD138, and EMA was found in our case and in some other reported cases [2, 3, 5, 6]. Moreover, tumors cells were positive for CD45, CD43, CD30, bcl2, BOB1, and OCT2 in some cases [2, 3, 5, 6].

PCR analysis revealed a polyclonal or oligoclonal pattern of Ig gene rearrangement, justifying the term of lymphoproliferative disorder used for this entity rather than lymphoma [1–7].

The presence of immunoblastic or plasmablastic morphology in a suspected hematolymphoid neoplasm should prompt investigation of a differential diagnosis that includes large B-cell lymphoma arising in HHV8-associated MCD and primary effusion lymphoma [11, 12]. In some cases, tumor cells may have an Hodgkin morphology mimicking lymphocyte-rich classical Hodgkin lymphoma or nodular lymphocyte predominant Hodgkin lymphoma [3].

In our case, the main differential diagnosis was an early stage of large B-cell lymphoma arising in HHV8-associated MCD due to the presence of atypical plasmablasts which coalesce to form nodules adjacent to or replacing some follicles. Furthermore, there were some regressive germinal centers, exhibiting features of Castleman disease in the adjacent lymph node parenchyma. Unlike GLD, MCD-associated large B-cell lymphoma arises in the setting of immunocompromised patients, often HIV+ with an aggressive clinical course. Furthermore, plasmablasts in MCD are CD20+ and EBV negative and show exclusively lambda light chain restriction [8, 10].

Primary effusion lymphoma often occurs in HIV patients which typically present with effusions in the absence of lymphadenopathy [10–12]. Given the clinical presentation in our case, the differential diagnosis is discussed with extracavitary variant of this affection, which shares similar morphology and immunophenotype features [12]. Histologic features showed that tumor cells are EBV+ and HHV8+ and lack B-cell antigens but differ from GLD by their more anaplastic morphology. Furthermore, surface and cytoplasmic expression of immunoglobulin are often absent with clonal Ig gene rearrangements in the PCR analysis [6, 8].

In GLD, plasmablasts may have an Hodgkin morphology and lesions can thus be misdiagnosed as lymphocyte-rich classical Hodgkin lymphoma or nodular lymphocyte predominant Hodgkin lymphoma, especially when germinotropism is not obvious (early forms). Immunophenotype is helpful in these cases, showing the coinfection HHV8/EBV [8].

The treatment included surgical excision associated or not with radiotherapy and/or chemotherapy.

Three patients were successfully treated with the conventional CHOP combination therapy (cyclophosphamide, vincristine, and prednisone) associated with rituximab in one case (R-CHOP) with a follow-up ranging from 10 months to 7 years [2, 3, 5].

After lymphadenectomy, cervical radiotherapy was administrated in 2 cases with a complete remission for 1 and 15 years, respectively [2, 6]. In 2 cases, the outcome was not available [2, 7].

As in our case, no additional therapy was done after the lymphadenectomy in 2 cases, with a favorable course after 2 and 7 years, respectively [1, 4]. This attitude raises questions about the accurate benefit of an adjuvant treatment in the management of this entity.

## 4. Conclusion

HHV8/EBV-associated germinotropic lymphoproliferative disorder is a rare disorder that has been described in HIV seronegative patients. It is an indolent disease which usually presents as an isolated nodal affection with a favorable outcome after treatment.

Morphological features include the presence of a large number of plasmablasts often involving germinal centers. This germinotropism may be absent, especially in early form, and should not eliminate the diagnosis, which requires the correlation with the clinical presentation.

It is a challenging diagnosis for the pathologists given the lack of expression of common lymphoma-associated markers and the main differential diagnosis includes large B-cell lymphoma arising in HHV8-associated MCD and primary effusion lymphoma. In some cases, tumor cells may have Hodgkin morphology mimicking lymphocyte-rich classical Hodgkin lymphoma or nodular lymphocyte predominant Hodgkin lymphoma. In these cases, the coinfection of tumor cells with EBV and HHV8 and the polyclonal or oligoclonal pattern of Ig gene rearrangement in molecular analysis are very suggestive features.

The treatment is not yet well codified but it is interesting to note the successful management of some cases treated only with simple lymphadenectomy without adjuvant therapy.

A long-term follow-up and larger number of cases are required for a better knowledge of this entity.

## Figures and Tables

**Table 1:** Summary of relevant clinicopathological features of the HHV8/EBV-associated GLD reported in the literature.

Authors	Sex/age (y)	Clinical presentation/viral serology	Pathologic features		ISH/PCR analysis	Therapy/Outcome
			Histology	IHC		
D'Antonio et al. [1]	M/60	Laterocervical lymphadenopathy Fever Fatigue and weight loss HIV: Neg	Partially effaced architecture Vaguely nodular infiltration of plasmablasts in the germinal centers, the mantle, and the interfollicular zones	*Positive IS:*HHV8, MUM1, kappa light chains, cytoplasmic IgM Ki67 (most cells) *Negative IS:*CD45, CD20, CD19, CD22, CD79a, CD10, Bcl2, Bcl6, CD30, CD138	EBER + Polyclonal pattern of Ig gene rearrangement	No adjuvant therapy CR for 2 y

Du et al. [2]	M/41	Axillary, cervical, and right perirenal lymphadenopathies HHV8+, EBV+ HCV+, HIV−	Preserved architecture germinal centers were replaced partially or completely by tumor cells: plasmablasts and anaplastic cells Normal mantle zones Prominent plasmacytosis in the interfollicular zones	*Positive IS:*HHV8+ *Negative IS:* CD20, CD27, CD30, CD38, CD79a CD138, bcl6, CD10	EBER+ Oligoclonal VH gene mutation	Six cycles of CHOP, CR for 7 y
	M/61	Submandibular, inguinal, and paratracheal lymphadenopathies HHV8+, EBV+, HIV−		*Positive IS:*CD30, CD38 *Negative IS:* CD20, CD27, CD79a, CD138 BCL6, CD10, EMA	EBER+ PCR failed	Cervical lymph node excision + RT CR for 15 y
	F/63	Left leg swelling and paraesthesia para-aortic lymphadenopathy Not available		Positive IS CD38 *Negative IS:*CD20, CD27, CD30, CD79a, CD138, BCL6, CD10, bcl2	EBER+ Polyclonal pattern of Ig gene rearrangement	Not available

Taris et al. [6]	F/49	Isolated jugular lymphadenopathy during 4 months VIH−, HBV−, HCV−, HTLV 1/2−	Nodular architecture Clusters of Hodgkin-like cells and plasmablasts in the hyperplastic mantle zones	*Positive IS:*EMA, MUM1, Bcl2, BOB1, OCT2, HHV8 CD38 (focal) Lambda light chains *Negative IS:* CD20, CD79a, CD3, PAX5, CD30, CD15, CD138, ALK	EBER + Polyclonal pattern of Ig gene rearrangement	Cervical lymphadenectomy + RT CR for 1 y

Ferry et al. [3]	M/61	Asymptomatic cervical, supraclavicular, paratracheal, and mediastinal lymphadenopathies discovered on the occasion of a ruptured aneurysm HIV−	Large and atypical cells in the interfollicular zones Rare apoptotic, mummified, and RS-like cells Some regressive follicles like those of Castleman disease	*Positive IS:*HHV8, CD45, EMA, MUM1/IRF4, CD138 *Negative IS:* CD15, CD30, CD20, Pax5, CD3, Alk cytokeratin	EBER+/monotypic lambda light chain + coexpression of *μ* heavy chain Polyclonal for IgH rearrangements	Six cycles of CHOP + rituximab CR for 10 months

D'Antonio et al. [4]	M/65	Right cervical lymph node HIV−	Partial architectural effacement Vaguely nodular infiltrate of plasmablasts/immunoblasts, involving the germinal centers	*Positive IS:*MUM1, kappa light chains, cytoplasmicIgM, vIL-6EBV, HHV8, KI67 (most cells) *Negative IS:* CD20 LCA, CD79a, CD10, bcl6, bcl2, CD30, CD43, CD138, CD15	Polyclonal Ig VH/ORF72+	No adjuvant therapy Alive 7 years

Oh et al. [5]	M/75	Incidental submandibular lymphadenopathy discovered in PET (health screening program) HIV−	Partially effaced architecture Solid aggregates of plasmablasts in the germinal centers	*Positive IS:*HHV-8, CD138, EMA, CD43, kappa light chain Negative: CD3, CD5, CD10, CD15, CD20, CD21, CD23, CD30, CD45 CD56, CD68, CD79a, vimentin	EBER+/polyclonal pattern for Ig heavy chain (IgH) gene rearrangement	Four courses of CHOP Disease-free for 19 months

Papoudou-Bai et al. [7]	M/53	Supraclavicular lymphadenopathy	Preserved lymph node architecture Germinal centers invaded by a proliferation of plasmablastic/immunoblastic cells	*Positive IS:*HHV8, MUM1/IRF4, CD38 *Negative IS:* CD30, CD15, CD45, CD20, CD79a, PAX5, CD5, CD2, CD4, CD8, cyclin D1, CD23, CD10, CD138, bcl6, LMP1	EBER+ Expression of *μ* heavy chain	Not available

Current case	F/78	Inguinal lymphadenopathies incidentally discovered (hepatic decompensation in a cirrhotic patient)	Partial effaced architecture Nodular lymphoid proliferation of plasmablastic cells in the germinal centers and the mantle zones Some regressive germinal centers	*Positive IS:*EMA, CD38, CD138, kappa light chain, HHV8+, LMP1 *Negative IS:* AE1/AE3, CD20, CD3, cyclin D1, CD56, CD15, CD30, CD10, bcl2	EBER+/monotypic lambda light chain Polyclonal pattern of Ig gene rearrangement	One inguinal lymphadenectomy Lost to view

PET: positron emission tomography, y: years, HCV: hepatitis C virus, HBV: hepatitis B virus, HTLV1: human T-cell lymphotropic virus, RS: Reed Sternberg, IHC: immunohistochemistry, IS: immunostaining, ISH: in situ hybridation, EBER: EBV encoded RNA, Ig: immunoglobulin, CHOP: cyclophosphamide, doxorubicin, vincristine, and prednisone, RT: radiotherapy, and CR: complete remission.
